# An Efficient Metaheuristic-Based Clustering with Routing Protocol for Underwater Wireless Sensor Networks

**DOI:** 10.3390/s22020415

**Published:** 2022-01-06

**Authors:** Neelakandan Subramani, Prakash Mohan, Youseef Alotaibi, Saleh Alghamdi, Osamah Ibrahim Khalaf

**Affiliations:** 1 Department of Computer Science and Engineering, R.M.K Engineering College, Chennai 601206, India; 2 Department of Computer Science and Engineering, Karpagam College of Engineering, Coimbatore 641032, India; salemprakash@gmail.com; 3 Department of Computer Science, College of Computer and Information Systems, Umm Al-Qura University, Makkah 21955, Saudi Arabia; yaotaibi@uqu.edu.sa; 4 Department of Information Technology, College of Computers and Information Technology, Taif University, Taif 21944, Saudi Arabia; s.algamedi@tu.edu.sa; 5 Department of Computer Engineering, Al-Nahrain Nano Renewable Energy Research Center, Al-Nahrain University, Baghdad 10071, Iraq; usama.ibrahem@coie-nahrain.edu.iq

**Keywords:** clustering, routing, energy efficiency, underwater wireless sensor network, metaheuristics, fitness function

## Abstract

In recent years, the underwater wireless sensor network (UWSN) has received a significant interest among research communities for several applications, such as disaster management, water quality prediction, environmental observance, underwater navigation, etc. The UWSN comprises a massive number of sensors placed in rivers and oceans for observing the underwater environment. However, the underwater sensors are restricted to energy and it is tedious to recharge/replace batteries, resulting in energy efficiency being a major challenge. Clustering and multi-hop routing protocols are considered energy-efficient solutions for UWSN. However, the cluster-based routing protocols for traditional wireless networks could not be feasible for UWSN owing to the underwater current, low bandwidth, high water pressure, propagation delay, and error probability. To resolve these issues and achieve energy efficiency in UWSN, this study focuses on designing the metaheuristics-based clustering with a routing protocol for UWSN, named MCR-UWSN. The goal of the MCR-UWSN technique is to elect an efficient set of cluster heads (CHs) and route to destination. The MCR-UWSN technique involves the designing of cultural emperor penguin optimizer-based clustering (CEPOC) techniques to construct clusters. Besides, the multi-hop routing technique, alongside the grasshopper optimization (MHR-GOA) technique, is derived using multiple input parameters. The performance of the MCR-UWSN technique was validated, and the results are inspected in terms of different measures. The experimental results highlighted an enhanced performance of the MCR-UWSN technique over the recent state-of-art techniques.

## 1. Introduction

Water covers the Earth in different ways, in the form of oceans, rivers, and lakes. It is important for humans and other animals to have water in their lives and for other animals to have water as well. Advances in technology have made it possible to place sensor nodes in lakes, river environments, and natural forests so that they can be used to study them. It is possible for these sensor nodes to communicate with each other because they have smart computing and smart sensors built in. Networks called underwater wireless sensor networks (UWSNs) are made up of a lot of autonomous sensors that are limited in energy and homogeneous nodes [1]. These underwater sensors were used to look for things such as pressure, temperature, water quality, and current flow in the water. They were placed in seas and rivers. Based on the types of applications, the data processing station gathers these kinds of data. A summary of how it works can be found below.

**Figure 1:**
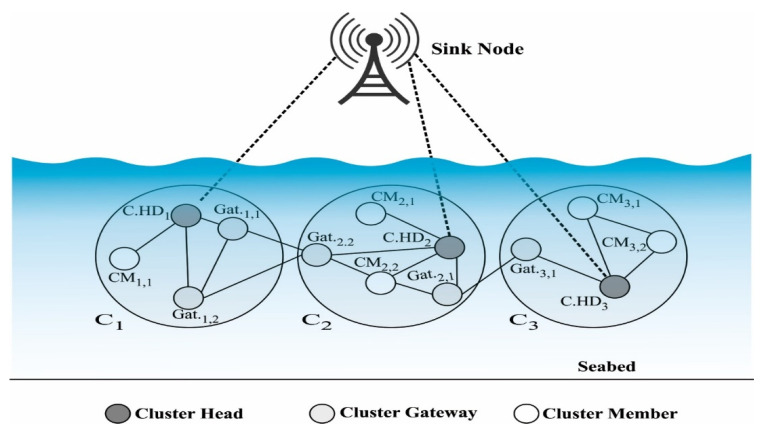
Overview of cluster based UWSN.

Infrastructure for underwater sensors is made up of different types of devices: an acoustic modem, a memory, and a sensor. They also have an on-board controller, a power supply, and a sensor interface circuitry. It is possible to use the underwater sensors to measure the density and temperature under the water, among other things. They could also be used to measure acidity, pH, conductivity, turbidity, hydrogen, and dissolved methane gas. [2] Figure 1 shows the network models used by UWSN. Onshore base station, underwater sensors, and sink node are all part of the UWSN (SN). The nodes were close to the SN and sent information to the sink, while another node made the clusters. Underwater sensors send information to the SN at the surface [3]. The data are then sent to a nearby base station (BS) [4]. One of the two types of transceivers that the SN nodes have is an acoustic transceiver that can communicate with the sensors. The other type is a radio transceiver that can communicate with the BSS using radio frequency. To send messages to other nodes, underwater nodes have an acoustic transceiver that is built into them. Communication in underwater environments is different from in terrestrial wireless sensor networks (TWSN) in terms of topology, channel modelling, and path loss. The UWSN is moved by current at a speed of about 1–3 m/s. There are different models for the acoustic and radio channels.

The minimum clash probability routing underwater wireless sensor networks (MCR-UWSN) is made up of many underwater acoustic sensors that are placed in underwater monitoring areas to carry out surveillance, navigation, intrusion detection, data collection, and resource exploration [4,5]. Another problem with the Ant Colony Optimization Clash probability Routing (ACOCR) UWSN is that it has a huge error rate, propagation delay, and a low bandwidth. It is very important for UWSN to come up with an energy-efficient way to send data in complicated underwater environments [6]. There are a lot of traditional ways to route in TWSN. However, in UWSN, they are almost always impossible.

Multi-hop data transmissions for UWSN in long-distance transmissions are much more efficient than single-hop data transmissions [7]. Furthermore, to help with traffic load balance and data collision, it is important to have a consistent network topology [8]. In a lot of studies, it has been shown that cluster routing algorithms are good at avoiding collisions. They balance traffic loads, and they use multi-hop mechanisms to send data between clusters [8]. There are many groups of nodes in a cluster routing algorithm, and each group has a head node (CHN) and a lot of nodes called “cluster member nodes”. As soon as clusters were made, the CHN allocates channels to send data through. The CMN sends data based on the distribution that might avoid collisions [9]. CHNs are then responsible for aggregation, which might reduce data redundancy and cut down on the number of data packets that need to be sent to the SN, which saves energy [10]. Clustering routing algorithms are better at cutting down on data transmission and managing traffic, according to a lot of research. They can also cut down on the number of packets lost, save energy, and keep the network running for a long time. The underwater sensors are restricted to energy and it is tedious to replace or recharge batteries, resulting in energy efficiency being a major challenge. Clustering and multi-hop routing protocols are considered energy-efficient solutions for UWSN. However, the cluster-based protocols for traditional wireless networks could not be feasible for UWSN owing to the underwater current, high water pressure, low bandwidth, propagation delay, and error rate. To resolve these issues and achieve energy efficiency in UWSN, this study focuses on designing the metaheuristics-based clustering with a routing protocol for UWSN, named MCR-UWSN. The goal of the MCR-UWSN technique is to select an optimal set of cluster heads (CHs) and route to destination.

The main contributions and novelty of this research are as follows.

(i) To solve the issue of clustering, multi-hop routing protocols are considered energy-efficient solutions for UWSN. However, the cluster-based routing protocols for traditional wireless networks could not be feasible for UWSN owing to the underwater current, low bandwidth, high water pressure, propagation delay, and error probability.

(ii) To resolve these issues and achieve energy efficiency in UWSN, this study focuses on designing metaheuristics-based clustering with a routing protocol for UWSN, named MCR-UWSN.

(iii) The MCR-UWSN method picks the best CHs and the shortest routes to the BS. MCR-UWSN is a technique that uses the cultural emperor penguin optimizer-based clustering (CEPOC) method to choose the best CHs and build groups.

(iv) The goal of the MCR-UWSN technique is to elect an efficient set of cluster heads (CHs) and route to destination. In addition, a multi-hop routing method that uses grasshopper optimization (MHR-GOA) is proposed in this paper.

(v) In order to show how the MCR-UWSN technique improves performance, a series of simulations are run and the results are looked at in different ways.

The rest of the paper is arranged in the following way. Section 2 explores the literature. Section 3 provides a model of the proposed system. This is how we came up with the CEPOC technique and the MHR-GOA technique. Section 4 details the experiments and shows the results. The given framework is shown to work well in this section. Section 5 finally concludes the paper.

## 2. Literature Review

This section provides a brief overview of UWSN’s existing cluster-based routing techniques. Nguyen et al. [11] developed a low-energy adaptive clustering hierarchy (LEACH) approach for balancing this node’s power utilization and enhancing the network’s lifespan. In regards to depth levels, the network regions are divided into layers. The data collected by the nodes are routed via multi-hop routing paths to an SN. The CHs are selected based on the node’s depth. The CH collects the information packets of each cluster member and then forwards them to the SN’s upper layer in order to send information from the nodes to the SN.

Khan et al. [12] presented a novel system for UWSN based on cooperative energy harvesting. The system employs the AF technique at the relay node to convey data and the FCR method at the end node to choose accurate signals. The presented technique chooses relay nodes from among its neighbor nodes based on the amount of energy gathered. Almost all of UWSN routing techniques based on cooperation do not include energy harvesting mechanisms at the relay nodes. EH-ARCUN incorporates piezoelectric energy harvesting at the relay node to increase the work capacity of sensor nodes in the UWSN with an energy-based cluster optimization algorithm.

Bhattacharjya et al. [13] design an energy-efficient UWSN capable of reducing energy costs and increasing efficiency in underwater settings. A UWSN framework is built in the provided cluster-based underwater wireless sensor network (CUWSNs), which takes use of the benefits of CH and multi-hop transmissions. The described CUWSN enhances a network lifespan by multi-hop broadcasts.

Zhu and Wei [14] offer the EERBLC method, a localization-free routing mechanism. EERBLC protocols are divided into three stages: cluster update and maintenance, transmission routing, and layer and uneven cluster construction. Initially, the monitoring regions under the sea were divided into levels, and nodes in comparable layers were grouped. A novel un-equal clustering strategy based on layer for UWSN is described in order to balance energy across whole networks and prevent “hotspot” concerns.

Sahana and Singh [15] provided a method for fuzzy-based CH selection that increases network longevity. Then, they created a fuzzy-based routing technique that significantly increases the packet transmission rate. In comparison to the current routing protocol, they have adequate power consumption and, hence, prolong the overall performance of the network for overcoming the problems underwater.

To protect against internal attacks, Fang et al. [16] describe an LTMS technique based on binomial distribution. Simultaneously, energy, distance, environment, and security domains are considered and presented to propose an MSCR system through dynamic dimension weight in hierarchical WSN.

Gomathi et al. [17] present a unique routing strategy known as the EE-MDCHSRP technique, which is suggested to the UWSN for successful dynamic CH choices. The dynamic CH node is defined by taking into consideration the residual energy factor, the least mobility factor, and the node density.

Sher et al. [18] describe three energy-efficient routing strategies for monitoring the field with circular and square geometries for underwater WSNs; they are sparsity-aware energy-efficient clustering based on circular depth, sparsity-aware energy-effective clustering routing protocol, and circular sparsity-aware energy-efficient clustering. Each protocol is designed to reduce the power needs of sparse areas, while density search techniques are used to locate dense regions and sparsity search algorithms are presented to find sparse network fields.

Karim et al. [19] suggested two network frameworks with numerous sinks: the VH-ANCRP technique for addressing local maximum nodes and the ANCRP approach for obtaining consistent data transmission metrics. To form clusters, the network areas are divided into smaller cubes. As a CH, all cubes are assigned using anchor nodes [20]. When a source node is freely allocated, each CH is considered to be anchored at the centroid of a cube through strings. In ANCRP, the source node is in charge of relaying sensed data to their chosen CHs.

The primary issues of this research are as follows. Recently, energy efficiency has emerged as a critical concern in wireless sensor networks. Sensor networks are powered by batteries and, as a result, die after a specific amount of time [21]. As a result, optimizing data dissipation in an energy-efficient manner becomes a more difficult task in order to increase the lifespan of sensor devices. Clustering and tree-based data aggregation for sensor networks can improve wireless sensor network lifespan. Clustering and multi-hop routing protocols are considered energy-efficient solutions for UWSN. However, the cluster-based routing protocols for traditional wireless networks could not be feasible for UWSN owing to the underwater current, low bandwidth, high water pressure, propagation delay, and error probability [22]. An energy-efficient clustering and tree-based routing protocol based on hybrid Ant colony optimization (ACO) and particle swarm optimization (PSO) is suggested (Supreet Kaur et al., 2018). To resolve these issues and achieve energy efficiency in UWSN, this study focuses on designing the metaheuristics-based clustering with a routing protocol for UWSN, named MCR-UWSN. The goal of the MCR-UWSN technique is to elect an efficient set of cluster heads (CHs) and route to destination. The MCR-UWSN technique involves the designing of cultural emperor penguin optimizer-based clustering (CEPOC) techniques to construct clusters. Besides, a multi-hop routing technique using grasshopper optimization (MHR-GOA) is derived with multiple input parameters [23].

## 3. The Proposed Model

This paper proposes a novel MCR-UWSN approach for UWSN energy efficiency. MCR-UWSN uses a two-stage process: CEPOC cluster creation and MHR-GOA routing. These procedures are described in depth in the following sections.

### 3.1. System Model

The network scenarios include N dynamic nodes that are sparsely and randomly distributed throughout a L × L × L. The data sources are the water medium’s sensed data. The data are collected using underwater sensors. Pressure, temperature, and current flow are all detected parameters. Underwater sensors are equipped with acoustic modems that allow them to communicate with another node submerged in the water [24]. The SN is located on the surface landmass and is equipped with RF and acoustic modems; the SN acoustic modem collects data from underwater sensors, while the RF modems transfer data to the BS. They assumed this network scenario was connected to the network. Due to the fact that the underwater sensors are movable due to the water current’s velocity of around 1–3 m/s, the topology changes rapidly [25]. The network’s assumption might be characterized as follows:

1. The nodes know its position and the position of SN on initial placement;
2. Nodes might become the CH, and clusters member/relay;
3. The CH is rotated among the sensors for conserving energy.

Due to the fact that the properties of acoustic waves in an underwater broadcast medium differ from those of radio waves, the power consumption of WSN cannot be utilized for UWSN. They apply the underwater acoustic channel’s power consumption strategy for the present investigation [26]. The energy used while transferring *k* bits of data across a distance *d* at a data rate *R* is calculated as follows.

(1) $$E_{Tx}\left(k,d\right)=k\times E_{elec}+\frac{k}{R}P_{tx}$$

where $E_{elec}$ represents the power consumptions to route one bit of data and $P_{Tx}$ means the transferred power [27].

For receiving k bit of data, the receiver radio’s power consumptions are given below

(2) $$E_{Rx}\left(k\right)=kP_{r}$$

Let $P_{r}$ denote a constant based on the device. For fusing k bit of data, the power consumptions are determined by

(3) $$E_{DA}\left(k\right)=k\times E_{DA0}\;$$

In which $E_{DA0}$ indicates the energy expended by fusing 1 bit of data, i.e., taken as five nJ/bit. As nodes are mobile because of the water currents, they place arbitrary motion for nodes during operational time. The present velocities are 1–3 m/s.

### 3.2. Design of CEPOC Technique

The CEPO’s central notion is to derive information about issue resolution from EPO’s budding behavior and to use that knowledge to guide EPO’s evolution concurrently. Assume CEPO is designed to solve situations with minimum optimization:

(4) $$\min\;f\left(x_{i}\right)$$

where $x_{i}=\left(x_{i1},x_{i2},\;\ldots ,x_{iD}\right)$ represents the location of *ith* EPO in the D-dimension search region; $x_{j}^{\min}<x_{ij}<x_{j}^{\;\max\;},\left(j=1,2,\;.\;.\;.\;,\;D\right)$. f( ) denotes the objective function; and $f\left(x_{i}\right)$ signifies the objective value of the location $x_{i}$. $x_{j}^{\min}$ and $x_{j}^{\max}$ represent the lower bound and upper bound of the location of emperor penguin in the jth dimensions, respectively [28].

**Figure 2:**
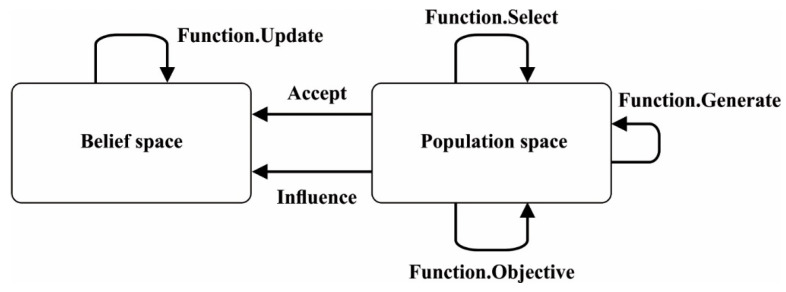
Workflow of CEPO algorithm.

The belief space of EPO population in the tth generation is determined as $s^{t}$ & $N_{j}^{t}$, in which $s^{t}$ implies situational knowledge components. $N_{j}^{t}$ denotes normative knowledge that represents the value space data for all parameters in the jth dimensions and tth generations. $N_{j}^{t}$ represents I, L, U. $I_{j}^{t}=[l_{j}^{t},\;u_{j}^{t}$], whereas $I_{j}^{t}$ denotes the interval of normative knowledge in jth parameter. The lower bound $l_{j}^{t}$ and upper bound $u_{j}^{t}$ are initiated based on the value range of parameters provided by the problems [29]. $L_{j}^{t}$ characterizes the objective value of lower bound $l_{j}^{j}$ of the jth dimension and $U_{j}^{t}$ represent the objective value of upper bound $u_{j}^{t}$ of the jth dimension. The acceptance functions are employed for selecting the EPO could directly impact the present belief space. In CEPO, the acceptance functions select the individual in proportion to 20% from the present population space for updating the belief space. Figure 2 illustrates the work flow of algorithm [30].

Situational knowledge $s^{t}$ could be upgraded with upgrade functions:

(5) $$s^{t+1}=\{\begin{array}{cc} x_{best}^{t+1} & \mathrm{if}\;f\left(x_{best}^{t+1}\right)<f\left(s^{t}\right)\\ s^{t}, & else,\; \end{array}$$

where $x_{best}^{t+1}$ represents the optimum location of EPO population space in the (t+1)th generation [31].

Consider that the qth cultural individual, an arbitrary parameter $\theta_{q}$ in the interval of zero and one, is generated. The qth cultural individual affect the lower bound of normative knowledge in the *j*th parameter if $\theta_{q}<0.5$ is fulfilled. Normative knowledge $N_{j}^{t}$ could be upgraded with upgrade functions:

(6) $$l_{j}^{t+1}=\{\begin{array}{cc} x_{qj}^{t+1}, & if\;x_{qj}^{t+1}\leq l_{j}^{t}\;or\;f\left(x_{q}^{t+1}\right)<L_{j}^{t}\;\\ l_{j}^{t}, & else, \end{array}$$

$$L_{j}^{t+1}=\{\begin{array}{cc} f\left(x_{q}^{t+1}\right), & if\;x_{qj}^{t+1}\leq l_{j}^{t}\;or\;f\left(x_{q}^{t+1}\right)<L_{j}^{t}\;\\ L_{j}^{t}, & else. \end{array}$$

The qth cultural individual affect the upper bound of normative knowledge in jth parameter if $\theta_{q}\geq 0.5$ is fulfilled:

(7) $$u_{j}^{t+1}=\{\begin{array}{cc} x_{qj}^{t+1}, & if\;x_{qj}^{t+1}\leq u_{j}^{t}\;or\;f\left(x_{q}^{t+1}\right)<U_{j}^{t}\;\\ u_{j}^{t}, & else, \end{array}$$

$$U_{j}^{t+1}=\{\begin{array}{cc} f\left(x_{q}^{t+1}\right), & if\;x_{qj}^{t+1}\leq u_{j}^{t}\;or\;f\left(x_{q}^{t+1}\right)<U_{j}^{t}\;\\ U_{j}^{t}, & else. \end{array}$$

Normative and situational knowledges could be employed for guiding EPO population development with the impact functions [32]. In CEPO, an election operator β is generated to affect the evolutional EPO population:

(8) $$\beta =\frac{{\mathrm{Max}}_{\mathrm{iteration}}-t}{{\mathrm{Max}}_{\mathrm{iteration}}}$$

where ${\mathrm{Max}}_{\mathrm{iteration}}$ represents the maximal numbers of iteration. Consider that ith EPO, an arbitrary parameter $\lambda_{i}$ in the interval of zero and one is generated [33]. The initial manner is to upgrade the location of EPO by altering the search size and direction of the variations using belief space that can be executed while fulfilled $\lambda_{i}\leq \beta$. The location of EPO in the jth parameter will be upgraded as follows

(9) $$x_{ij}^{t+1}=\{\begin{array}{cc} x_{ij}^{t}+\left|\mathrm{size}\left(I_{j}^{t}\right)\cdot N\left(0,1\right)\right|, & if\;x_{ij}^{t}<s_{j}^{t},\\ x_{ij}^{t}-\left|\mathrm{size}\left(I_{j}^{t}\right)\cdot N\left(0,1\right)\right|, & if\;x_{ij}^{t}>s_{j}^{t},\\ x_{ij}^{t}+\eta \cdot \mathrm{size}\left(I_{j}^{t}\right)\cdot N\left(0,1\right), & else, \end{array}$$

where N(0, 1) represents an arbitrary value subject to the standard distribution [34]. The size $\left(I_{j}^{t}\right)$ means the length of adaptable range of jth dimension in belief space in tth generation. *η* is fixed between the interval [0.01, 0.6].

Another approach is using a sequence of phases in EPO, i.e., the temperature profile around the huddle computing, the huddle boundary generation, the position update of emperor penguins, and the distance calculation between emperor penguins, which would be brought while fulfilled $\lambda_{i}>\beta .$ The certain phases could be denoted in the following equation:

(10) $$T^{\prime }=T-\frac{t-{\mathrm{Max}}_{\mathrm{iteration}}}{{\mathrm{Max}}_{\mathrm{iteration}}}$$

$$T=\{\begin{array}{cc} 0, & \mathrm{if}\;R\geq 0.5\\ 1, & \mathrm{if}\;R<0.5 \end{array}$$

where $T^{\prime }$ denotes the temperature profile around the huddle, T indicates the time to find an optimum solution, and R signifies an arbitrary parameter in the interval of zero and one.

(11) $$D_{\mathrm{ep}}^{t}=S_{\mathrm{ep}}\left(A^{t}\right)\cdot x_{\mathrm{best}}^{t}-B^{t}\cdot x_{i}^{t}$$

where $D_{\mathrm{ep}}^{t}$ indicates the distance between the EPO and optimum solutions, $x_{\mathrm{best}}^{t}$ denotes the present optimum solution attain in EPO population space in the tth generation, $S_{\mathrm{ep}}$ represent the social force of the EPO which is accountable for convergence towards the best solution, $A^{t}$ & $B^{t}$ are employed for avoiding the collisions between adjacent EPO, and $B^{t}$ denotes an arbitrary parameter in the interval of zero and one [35]. $A^{t}$ could be calculated by:

(12) $$\begin{array}{l} A^{t}=\left(M\times \left(T^{\prime }+P_{\mathrm{grid}}^{t}\left(Accuracy\right)\right)\times rand\;\left(\right)\right)-T^{\prime }\\ P_{\mathrm{grid}}^{t}\left(Accuracy\right)=\left|x_{\mathrm{best}}^{t}-x_{i}^{t}\right| \end{array}$$

where M denotes the motion variable that holds a gap among EPOs for avoiding collisions and $P_{\mathrm{grid}}^{t}$ (Accuracy) determines the accurate variance by relating the differences between the EPOs. $S_{\mathrm{ep}}\left(A^{t}\right)$ in Equation (11) is calculated by:

(13) $$S_{\mathrm{ep}}\left(A^{t}\right)=\sqrt{\varepsilon \cdot e^{-t/\rho }-e^{-t}}$$

where e is the base of natural logarithm. ε & ρ represent the 2 control variables for improved exploitation and exploration within the interval of [1.5, 2] and [2, 3], respectively. Finally, the location of EPO is upgraded by:

(14) $$x_{i}^{t+1}=x_{\mathrm{best}}^{t}-A^{t}\cdot D_{\mathrm{ep}}^{t}.\;$$

In the CEPO algorithm, a M×N grid of sensor nodes can be generated in a geographical area [36]. Each sensor in a mesh network is assigned a unique ID, and these sensors are referred to as penguins. On the basis of this penguin, a search space is formed. The distance between each node is calculated and stored in a matrix using Euclidean distance [37]. The search space is parameterized by the dimension, as well as the lower and upper limits. Each penguin’s fitness value is determined by its location in the search space. The fitness matrix is used to track each penguin’s fitness level. Due to the repetitive nature of this process, the estimated fitness value is stored in a matrix on each iteration, and such matrices supply penguins with a low fitness value. By integrating the fitness and location values of penguins, ideal scores are produced, and the penguins’ positions are updated based on these scores [38]. As a result, this converges to the optimum solution via the use of reduction factors and acquires the ideal cluster required for effective communication based on the assumed parameters [39]. Following cluster formation, the next step is CH election. As a result, the following variables are used: grid size, node density, and node broadcast range. These variables correspond to the weights assigned in FF. An FF is calculated for the purpose of selecting the optimum solution from among the candidate solutions. An FF is critical to the strategy. Selecting the optimal CH lengthens the life of clusters and may aid network energy conservation. The following approach is used to calculate fitness values.

(15) $$Fitness\;function=w_{1}\times a_{1}+w_{2}\times a_{2}$$

where $a_{1}$ & $a_{2}$ describes the $delta_{difference}$ and $distance_{neighbor}$, respectively.

In Equation (15), $delta_{difference}$ represents the difference, and $distance_{neighbor}$ denotes the average distance of node. $delta_{difference}$ is employed as a condition for LB. $w_{1}$ indicates the weight assigned to $delta_{difference}$ and $w_{2}$ means the weight assigned to $distance_{neighbor}$. In certain cases, each cluster might contain equivalent number of nodes; however, in a real-time situation, it is not easier because of the sensor’s location variations due to water current and another impact $delta_{difference}$ is employed to measure the difference from an ideal degree to motion of a node from its neighbor. It is computed by:

(16) $$delta_{difference}=abs\left(ideal_{deg}-node_{deg}\right)$$

As a result of the current study’s findings that static CH election conditions increase the likelihood that a single variable will skew the FF and cause an incorrect CH election, the presented solution dynamically assigns weights to each parameter based on its negative impact on the FF and situations [40]. The starting values of all parameters are normalized between 0 and 10 in this approach, and the deviations of all parameters are calculated as follows:

(17) Dev(p)=[mean−parameter(p)]

The overall weight should be equivalent to one. Every node’s fitness value is defined in Equation (15), in which the values of parameters are employed and weight allocated to all the parameters.

### 3.3. Design of MHR-GOA Technique

Grasshoppers are classified as pests based on the damage they inflict on vegetation and crops. Rather than acting independently, the grasshopper organizes the world’s largest swarms. Individuals’ influence on a wind, swarm, food supply, and gravity all have an effect on swarm motion [41]. The GOA is a novel metaheuristic approach based on SI that is triggered by the greater range and abrupt movement of adult grasshoppers in a group as shown in Algorithm 1. The metaheuristic algorithm breaks the search technique into stages of exploitation and exploration.
**Algorithm 1:** Pseudo code of GOAInitialize $\;\;\;\;\;\;\mathrm{Start}\;\mathrm{the}\;\mathrm{swarms}\;X_{i}\left(i=1,2,\ldots ,n\right)$, Initiate cmax, cmin and maximal amounts of iteration; Evaluate the fitness of every search agent; T = optimal search agent; while (l ≤ Max amounts of iteration)  Upgrade c;  for every search agent    Regulate the distance amongst grasshopper in [1,4];    Upgrade the location of the present search agent;    Bring the present search agent back when it drives outside the boundary;  end for
      Upgrade T when it has an optimal solution;         l=l+1; end while return T; End

The grasshopper’s greater range and abrupt movements indicate the exploration stage, whereas the grasshopper’s limited movements indicate the exploitation stage. Mirjalili et al., provides a numerical module for this behavior, which may be denoted as:

(18) $$x_{i}=S_{i}+G+A$$

where $x_{i}$ denotes the location of i grasshopper, $S_{i}$ indicates the social interaction in a group, G represents the force of gravity performing on i grasshopper, and A signifies the wind direction. By extending $S_{i}$, G & A in (1), the formula is given by:

(19) $$x_{i}=\overset{N}{\underset{j=1,j\neq i}{\sum }}s\left(\left|x_{j}-x_{i}\right|\right)\frac{x_{j}-x_{i}}{d_{ij}}-g{\hat{e}}_{g}+u{\hat{e}}_{w}$$

where $s\left(r\right)=fe^{-r/l}-e^{-r}$ denotes the function which stimulates the influence of social interaction and N represents amount of grasshopper. $g{\hat{e}}_{g}$ indicates the extended G element, while g signifies gravitational force and ${\hat{e}}_{g}$ denotes unit vector directing to the center of the earth. $u{\hat{e}}_{w}$ represents the extended A element, u denotes the constant drift, and ${\hat{e}}_{w}$ indicates the unit vector directing towards the wind direction. $d_{ij}$ is the distance between the ith & jth grasshopper and is estimated as:

$$d_{ij}=\left|x_{j}-x_{j}\right|$$

Because grasshoppers swiftly locate their comfortable zone and exhibit poor convergence, the effects of wind and gravity are negligible in comparison to the interaction between grasshoppers [42]. This indicates that the numerical module must be modified as follows:

(20) $$x_{i}=c\left(\overset{N}{\underset{j=1,j\neq i}{\sum }}c\frac{\mathrm{ub}-\mathrm{lb}}{2}s\left(\left|x_{j}-x_{i}\right|\right)\frac{x_{j}-x_{i}}{d_{ij}}\right)+{\hat{T}}_{d}$$

where ub & lb represents the upper and lower boundaries of the search space, respectively; $T_{d}$ indicates value comparative to the target (optimal solution establish until now); and c denotes the reducing coefficient which balances the process of explorations and exploitations, that is denoted by:

(21) $$c=c_{\max}-iter\;\frac{c_{\max}-c_{\min}}{{\mathrm{Max}}_{\mathrm{iter}}}$$

where $c_{\max}$ denotes the maximal value (equivalent to one), $c_{\min}$ represents the minimal value (equivalent to 0.00001), iter indicates the present iteration, and ${\mathrm{Max}}_{\mathrm{iter}}$ signifies the maximum number of iterations. The primary objective of MHR-GOA is to find a novel route from CHS to BS [43]. The novel paths are recognized with the help of MHR-GOA as FF metric i.e., comprised of NDE, RE, and DTBS.

Initially, each FF defines appropriate solution to the executed problem. In routing, each FF indicates the transmission path in the cluster head to the SN. The significance of FF is associated with network locations of CH which are included in the SN [44]. The supremacy of FF is related to m+1, in which m indicates the amount of CH contained from the system. Where $F_{i}=\left(F_{i,1}\left(t\right),\;F_{i,2}\left(t\right)\ldots F_{i,m+1}\left(t\right)\right)$ is the *ith* FF and the position $F_{i,d},\forall_{i}1\leq \;i\leq m+1,\;\forall_{d}1\leq \;d\leq m+1\;$defines next-hop for sending data to BS. It is possible to be extremely focused on discovering the ideal path from CH to SN. It may be achieved with the use of FF in a variety of sub-objectives, such as NDE, RE, and Euclidean distance, among other things [45]. In order to convey data, each subsequent hop obtains the data and transfers it to the BS server. As a result, the highest possible RE of the following hop is given first priority. Furthermore, for the main sub-objective of using RE, f1 is enhanced as follows:

(22) $$f1=\overset{m}{\underset{i=1}{\sum }}E_{CHi}$$

Euclidean distance may be defined as the distance between CH and the following hop, as well as the distance between CH and SN. When distances are kept to a minimum, the energy consumption rate is likewise decreased. The following purpose is to reduce the distance between CHs and SN as measured in the following manner:

(23) $$f2=\frac{1}{\sum_{i=1}^{m}\mathrm{dis}\left({\mathrm{CH}}_{i},\mathrm{NH}\right)+\mathrm{dis}\left(\mathrm{NH},\mathrm{BS}\right)}$$

ND denotes the list of nodes in the next hops. If hop is composed of a small number of CH members, it consumes low energy in acquiring information from the nearby node and survives for a long period of time. Following that, the next-hop with a restricted node degree is notably picked. Finally, NDE is defined based on the degrees of node f3,

(24) $$f3=\frac{1}{\Sigma_{i=1}^{m}I_{i}}$$

As demonstrated in Equation (25), the weighted sum models are then processed for all sub-objectives and turned into a single objective model. Here $\alpha_{1},\alpha_{2}$ & $\alpha_{3}$ denotes the weight allocated to each sub objectives, where $\alpha_{i}\varepsilon \left(0,1\right)$ and $\alpha_{1}+\alpha_{2}+\alpha_{3}=1.$

(25) $$Fitness=\alpha_{1}\left(f1\right)+\alpha_{2}\left(f2\right)+\alpha_{3}\left(f3\right)$$

## 4. Performance Validation

**Figure 3:**
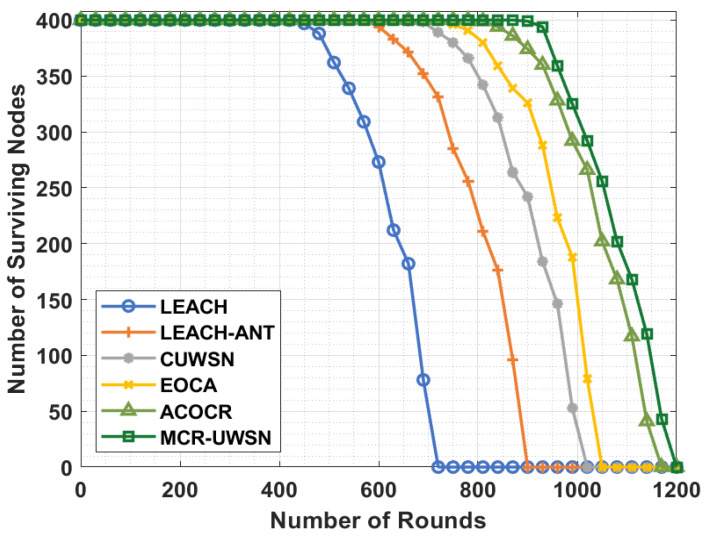
NSN analysis of MCR-UWSN method with existing approaches.

This section investigates the performance of the proposed MCR-UWSN technique with other techniques [46]. Figure 3 shows the network lifetime results of the MCR-UWSN model, such as the number of surviving nodes (NSN).

Figure 3 depicts that the LEACH technique has attained an ineffective performance with the least NSN. At the same time, the LEACH-ANT technique has gained slightly enhanced NSN over the LEACH technique. Besides, the CUWSN, EOCA, and ACOCR techniques have resulted in a moderate performance over the other techniques. However, the proposed MCR-UWSN technique has accomplished the superior performance with the maximum NSN.

**Figure 4:**
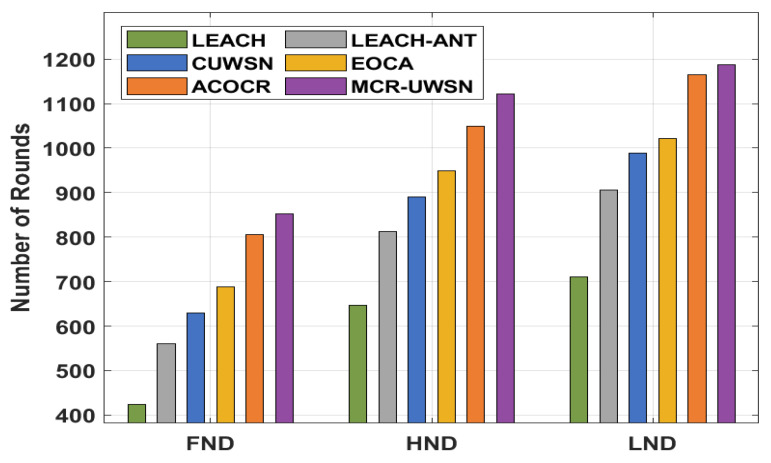
Result analysis of MCR-UWSN method with different measures.

Table 1 and Figure 4 illustrate the network lifetime analysis of the MCR-UWSN technique with existing techniques. From the figure, it is apparent that the MCR-UWSN technique has offered the maximum network lifetime [47]. With respect to FND, the MCR-UWSN technique has attained a higher FND of 852 rounds, whereas the LEACH, LEACH-ANT, CUWSN, EOCA, and ACOCR techniques achieved a lower FND of 424, 560, 629, 689, and 805 rounds, respectively. Moreover, in terms of HND, the MCR-UWSN approach reached a superior HND of 1121 rounds, whereas the LEACH, LEACH-ANT, CUWSN, EOCA, and ACOCR manners had an inferior HND of 646, 813, 891, 949, and 1050 rounds, respectively. Furthermore, with respect to LND, the MCR-UWSN method has the superior LND of 1187 rounds, whereas the LEACH, LEACH-ANT, CUWSN, EOCA, and ACOCR methods have a minimal LND of 710, 906, 989, 1021, and 1187 rounds, respectively.

**Figure 5:**
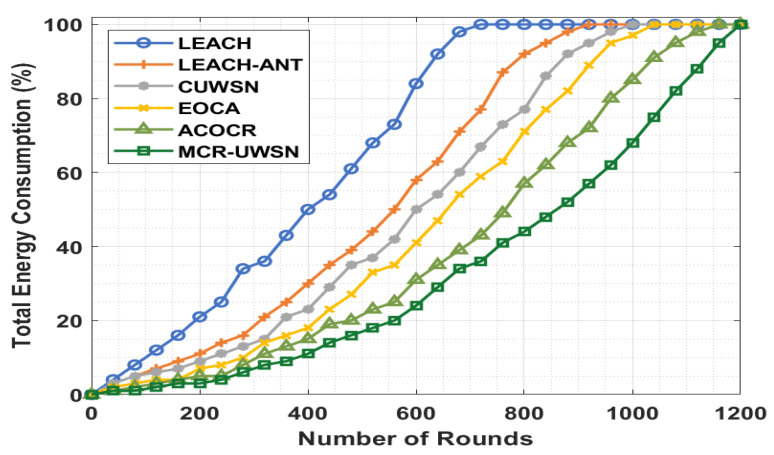
TEC analysis of MCR-UWSN method under distinct rounds.

Figure 5 demonstrates the total energy consumption (TEC) analysis of the MCR-UWSN technique with existing techniques. The figure depicts that the LEACH technique has gained an ineffective outcome with the higher TEC over the other techniques. Likewise, the LEACH-ANT technique has attained an improved performance, whereas the CUWSN, EOCA, and ACOCR techniques have a moderate TEC [48]. However, the MCR-UWSN technique has resulted in the superior performance over the other techniques with minimal TEC.

**Figure 6:**
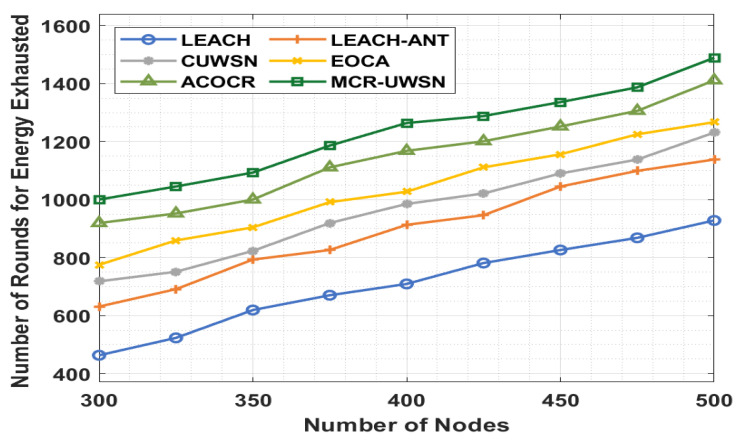
NREE analysis of MCR-UWSN method with existing approaches.

Table 2 and Figure 6 illustrate the network lifetime analysis of the MCR-UWSN technique with respect to the number of rounds for energy exhausted (NREE). The figure shows that the LEACH approach has an ineffective performance with the minimum NREE. Simultaneously, the LEACH-ANT method has a slightly higher NREE over the LEACH algorithm. In addition, the CUWSN, EOCA, and ACOCR methods resulted in a moderate performance over the other methods. Eventually, the presented MCR-UWSN method has accomplished a higher performance with the maximal NREE.

**Figure 7:**
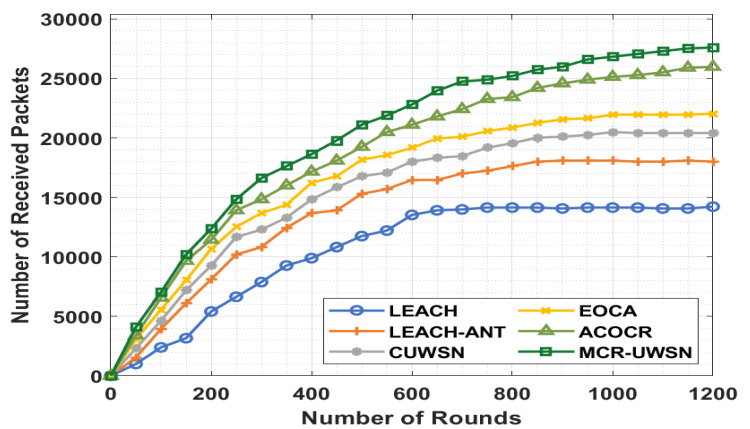
NRP analysis of MCR-UWSN method with existing approaches.

Figure 7 depicts the network lifetime analysis of the MCR-UWSN approach with respect to the number of received packets (NRP). The figure displays that the LEACH method has achieved an ineffective performance with the minimal NRP. Likewise, the LEACH-ANT method has reached slightly increased NRP over the LEACH method. Followed by the CUWSN, EOCA and ACOCR methods have a moderate efficiency over the other algorithms [49]. Finally, the projected MCR-UWSN algorithm has accomplished the maximal performance with a higher NRP.

The major strength of the proposed systems is that the MCR-UWSN approach consists of two stages: CEPOC-based cluster building and MHR-GOA-based routing. The CEPOC approach has produced a fitness function with unique input parameters for CH selection and cluster formation. It divides the sensor network into several parts, known as clusters, and cluster heads are selected in each cluster. Then, using short-distance connections, tree-based data aggregation takes over and collects sensory information directly from cluster heads. The CEPOC optimization determines the shortest path between the sink and the cluster heads. The use of compressive sensing minimizes the size of the packets that will be broadcast in the sensor network. A weakness of the proposed system is the lack of performance in the data aggregation process and underwater object tracking techniques. To address these concerns in the future, hybrid protocols will be utilized to extend the network lifespan by conserving energy more efficiently through data aggregation and object tracking for sensor networks.

## 5. Conclusions

In this study, a new MCR-UWSN technique is derived to accomplish an energy-efficient performance in UWSN. The MCR-UWSN technique incorporates a two stage process, namely CEPOC-based cluster construction and MHR-GOA-based routing. The CEPOC technique has derived a fitness function involving distinct input parameters for CH selection and cluster construction. Moreover, the MHR-GOA technique is proposed to optimally derive the routes for multi-hop communication. The MCR-UWSN method has the superior LND of 1187 rounds, whereas the LEACH, LEACH-ANT, CUWSN, EOCA, and ACOCR methods have a minimal LND of 710, 906, 989, 1021, and 1187 rounds, respectively. In order to prove an enhanced performance of the MCR-UWSN technique, a series of simulation processes take place, and the results are examined under different dimensions. The simulation results guaranteed an enhanced energy-efficient performance of the MCR-UWSN technique over the existing techniques. In the future, data aggregation and underwater object tracking techniques can be designed for UWSN.

## Figures and Tables

**Table 1:** Network lifetime analysis of the MCR-UWSN model with different measures.

Number of Rounds						
	LEACH	LEACH-ANT	CUWSN	EOCA	ACOCR	MCR-UWSN
FND	424	560	629	689	805	852
HND	646	813	891	949	1050	1121
LND	710	906	989	1021	1165	1187

**Table 2:** Result analysis of MCR-UWSN model in terms of the number of rounds for energy exhausted (NREE).

Number of Rounds for Energy Exhausted (NREE)						
Number of Nodes	LEACH	LEACH-ANT	CUWSN	EOCA	ACOCR	MCR-UWSN
300	463	631	718	775	919	1000
325	523	691	751	859	952	1045
350	619	793	823	904	1000	1093
375	670	826	919	991	1111	1186
400	709	913	985	1027	1168	1264
425	781	946	1021	1111	1201	1288
450	826	1045	1090	1156	1252	1336
475	868	1099	1138	1225	1306	1387
500	928	1138	1231	1267	1411	1489

## Data Availability

The study did not report any data.
